# Diabetic Nephropathy Induced by Increased *Ace* Gene Dosage Is Associated with High Renal Levels of Angiotensin (1–7) and Bradykinin

**DOI:** 10.1155/2015/674047

**Published:** 2015-09-09

**Authors:** Nádia Bertoncello, Roseli Peres Moreira, Danielle Yuri Arita, Danielle S. Aragão, Ingrid Kazue Mizuno Watanabe, Patricia S. Dantas, Ralmony Santos, Rodolfo Mattar-Rosa, Rodrigo Yokota, Tatiana Sousa Cunha, Dulce Elena Casarini

**Affiliations:** ^1^Nephrology Division, Department of Medicine, Federal University of São Paulo, Rua Botucatu 740, Vila Clementino, 04023-900 São Paulo, SP, Brazil; ^2^Science and Technology Department, Federal University of São Paulo, São José dos Campos, SP, Brazil

## Abstract

Population studies have shown an association between diabetic nephropathy (DN) and insertion/deletion (I/D) polymorphism of the angiotensin-converting enzyme (ACE) gene (*ACE* in humans, *Ace* in mice). The aim was to evaluate the modulation of *Ace* copies number and diabetes mellitus (DM) on renal RAS and correlate it with indicators of kidney function. Increased number of copies of the *Ace* gene, associated with DM, induces renal dysfunction. The susceptibility to the development of DN in 3 copies of animals is associated with an imbalance in activity of RAS enzymes leading to increased synthesis of Ang II and Ang-(1–7). Increased concentration of renal Ang-(1–7) appears to potentiate the deleterious effects triggered by Ang II on kidney structure and function. Results also show increased bradykinin concentration in 3 copies diabetic group. Taken together, results indicate that the deleterious effects described in 3 copies diabetic group are, at least in part, due to a combination of factors not usually described in the literature. Thus, the data presented here show up innovative and contribute to understanding the complex mechanisms involved in the development of DN, in order to optimize the treatment of patients with this complication.

## 1. Introduction

According to recent studies, 382 million people have diabetes and by 2035 this will rise to 592 million, posing the disease as one of the most challenging health problems of the 21st century [1, 2]. Diabetic nephropathy (DN) is a serious complication of diabetes and is the leading cause of end-stage renal disease (ESRD), accounting for approximately 40% of all cases [1, 3].

The renin angiotensin system (RAS) plays a key role in both renal physiology and the pathogenesis of chronic kidney disease and several studies to date have provided compelling support for the existence of an independent renal RAS whose activation appears to be critical in the development of DN [4, 5]. Angiotensin II (Ang II) is generated at higher levels in the kidney than in the systemic circulation and angiotensin converting enzyme 2 (ACE2), mainly related to angiotensin (1–7) [Ang-(1–7)] generation, is highly expressed in the kidney. It has been extensively described that activation of the RAS is associated with glomerular and tubular cell injury in chronic kidney diseases, including DN [6]. Within kidney cells Ang II stimulates a variety of signaling pathways linked to increased cell growth and production of extracellular matrix proteins, via binding to its AT1 receptor. In mesangial cells, binding of Ang II to AT1 receptors stimulates protein synthesis, cell hypertrophy, and the production of the profibrotic cytokine transforming growth factor, which is thought to be a critical mediator of progressive glomerulosclerosis [7].

On the other hand, it has been suggested that Ang-(1–7) is an important counterbalancing mechanism within the RAS having vasodepressor and antihypertensive actions in hypertensive animals or humans [8, 9]. Studies support a protective role for Ang-(1–7); this peptide prevents Ang II induced MAPK activation in proximal tubular cells [10] and its infusion has been reported to attenuate experimental glomerulonephritis [11]. Indeed, Giani et al. [8] showed that Ang-(1–7) reduced proteinuria and renal fibrosis in hypertensive rats. Similarly, Zhang et al. [11] found that Ang-(1–7) infusion attenuated glomerulosclerosis in rats and more recently attenuated kidney injury in mice with type 2 diabetes mellitus [12]. However, other studies suggest that some of the cellular effects of Ang-(1–7) may be deleterious; for example, stimulation of growth factor expression and cell proliferation by angiotensin in MC have been reported [13]. In addition, deletion of the gene for the Mas receptor in mice is associated with decreased inflammation in kidneys subjected to unilateral ureteral obstruction [14]. In rats with streptozotocin (STZ) induced diabetes, Benter et al. [15] have reported that chronic Ang-(1–7) treatment attenuated proteinuria and restored vascular reactivity. In contrast, Shao et al. [16] recently showed that chronic administration of Ang-(1–7) caused an increase in kidney TGF-*β*1 mRNA and protein, associated with increased proteinuria and renal injury in STZ-diabetic rats.

Although hyperglycemia is an important factor, it does not account fully for the risk of DN and alone it is not causative. A significant proportion of patients with diabetes do not develop DN despite long-standing severe hyperglycemia indicating that other factors, apart from chronic hyperglycemia* per se*, may equally contribute susceptibility for the development of DN. The familial aggregation of the DN demonstrates the involvement of genetic factors in the pathogenesis of this complication, and available data provide convincing evidence that the angiotensin converting enzyme I/D (*ACE* I/D) polymorphism is significantly associated with overt nephropathy being the insertion/insertion (II) genotype and low plasma ACE levels protective against the disease both in type I and type II diabetics [17–19]. These associations suggest that a high constitutive level of ACE is deleterious to the kidney in the setting of chronic hyperglycemia. However, a causal link between higher levels of ACE, angiotensin peptides, and kidney malfunction in association with diabetes has not been completely established.

Determining the contribution of a single gene to a complex phenotype such as DN remains a challenge since this is a context dependent response. The generation of genetically engineered mice with discrete lower and above normal ACE levels [20] overcomes some of these limitations, enabling, under controlled genetic and environmental conditions, testing the role* Ace* gene plays in DN. Huang et al. [21] showed that after 12 weeks of STZ induced diabetes, the 3 copies diabetic mice developed higher blood pressure and proteinuria, proving that a modest genetic increase in ACE levels is sufficient to initiate the chain of events leading to DN. The current study has extended these observations by characterizing the role of bradykinin and angiotensin peptides in face of increased and decreased dosage of* ACE* in the pathogenesis of diabetic lesions. Our results suggest that* Ace* locus associated with hyperglycemia disrupt RAS balance, generating a complex novel set of peptides synthesis. This includes not only a markedly increase on renal Ang II generation but also high tissue levels of bradykinin and Ang-(1–7), which in turn may have an important contribution on the development of diabetic lesions associated with increased* Ace* gene dosage.

## 2. Materials and Methods

### 2.1. Generation of Mice with 1 and 3 Copies of the* Ace* Gene

Mice (8–10 weeks old) were kindly provided from the Laboratory of Experimental Hypertension and Molecular Cardiology of University of São Paulo. Genetically engineered mice carrying either an inactivation (1 copy) or duplication (3 copies) of the* Ace* gene at its endogenous locus [20] were used for the study. Heterozygous mice of each strain were crossed for eight generations with wild-type C57BL6 mice and among themselves to generate the experimental groups. Identification of genetically modified offspring was made at 21 days of age by PCR amplification of DNA isolated from ear biopsies as previously described [22]. Animals were fed with standard laboratory chow and given water* ad libitum* while housed (4-5 per cage) in a temperature-controlled room (22°C) with a dark-light cycle of 12-12 h. All experimental procedures followed institutional guidelines for care, use, and euthanasia of laboratory animals, and the protocols were approved by the Institutional Review Board of Federal University of São Paulo, Brazil (1861/11). Efforts were exerted to reduce the number of animals used.

### 2.2. Experimental Procedure

Male mice of each genotype fasted 6 hours and then received a single intraperitoneal injection of STZ (150 mg/kg body wt. Sigma Chemical Company, St. Louis, MO, USA). The age-matched control mice were injected with an equivalent volume of saline. One week after STZ injection, blood samples were collected through the tail vein, and plasma glucose level was determined (Accu-Check Advantage Blood Glucose Monitor, Roche Diagnostic Corporation, Indianapolis, IN). Mice with plasma glucose ≥250 mg/dL and symptoms of polyuria, polyphagia, and polydipsia were considered to be diabetic and used in the present study. After the onset of diabetes, mice were monitored every other week for body weight and blood glucose, and every 4 weeks mice were placed in metabolic cages (Tecniplast, USA) for the determination of water and food intake, albumin, creatinine, and urea excretion in 24-hour urine output over a period of twelve weeks, without insulin treatment. At 12 weeks, 3 days after the last urine collection, mice were euthanized by decapitation and trunk blood was collected in ice-chilled heparinized tubes. Blood was centrifuged at 10,000 g for 10 minutes at 4°C. Plasma was separated, aliquoted, and stored at −80°C. Kidneys were rapidly excised, weighted, and processed for several purposes. Left kidney was frozen in liquid nitrogen for biochemical analysis (kept at −80°C until its use) and right kidney was fixed by immersion in formalin solution 10%, neutrally buffered, and processed for paraffin embedding according to standard procedures (for histological studies).

### 2.3. Metabolic Analysis

Mice were placed in metabolic cages for 24 h for the determination of urine excretion, water, and food intake. Albuminuria, urinary urea, and creatinine were quantified using respective enzyme-linked immunosorbent assay (ELISA, Mouse Albumin ELISA Quantification Set, Bethyl Laboratories, USA) and colorimetric commercially available kits (Labtest Diagnostic, Brazil). Metabolic analyses were performed at monthly intervals, during the 3-month protocol period. Incomplete data of metabolic parameters due to death of animals was deleted, and finally the total metabolic data of 6–11 mice in each group was recorded.

### 2.4. Tissue Preparation

At 12 weeks, 3 days after the last metabolic cage, animals were decapitated and trunk blood was collected. Kidneys were then removed, weighted, and processed for several purposes. Left kidney was rapidly excised, frozen in liquid nitrogen for biochemical analysis, and kept at −80°C until its use. The tissue was homogenized as described by Oliveira et al. [23] with some modifications related to tissue weight and buffer volume (12.5 mg kidney tissue/500 *μ*L borate buffer 0.4 M). The protein concentration was determined by the Bradford method [24] (Bio Rad Protein Assay Kit; Bio Rad, Hercules, CA) using bovine serum albumin as the standard. Right kidney was fixed by immersion in formalin solution 10%, neutrally buffered, and processed for paraffin embedding according to standard procedures for histological studies. So kidney slices, with a thickness of 5 mm, were stained using Hematoxylin-Eosin (HE), Periodic Acid Schiff Stain (PAS), and Masson's Trichrome in order to analyze the presence of renal lesions related to diabetes, or any other morphological changes. Analyses were conducted by a blinded observer.

### 2.5. Evaluation of Renin Angiotensin System

#### 2.5.1. Renal Renin

Active renin content was measured by determining the amount of angiotensin I (Ang I) generated in the tissue homogenates measured by high-performance liquid chromatography (HPLC), as previously described [25]. Active renin was evaluated in the absence of trypsin and to prevent further cleavage of angiotensinogen, and Ang I, a pool of enzymatic inhibitors, was added to the tissue homogenate: 50 Mm ethylenediaminetetraacetic acid (EDTA), 1 mM 1,10-phenanthroline (OPhe), 3 mM phenylmethanesulfonyl fluoride (PMSF), and 200 mM dithiothreitol (DTT). Renin activity was estimated by Ang I generation when the tissue homogenate was incubated with 10 *μ*L of 2 nmol/mL synthetic tetradecapeptide substrate (Sigma, St. Louis, MO) for 1 h at 37°C, as previously described [26]. The reaction was stopped at two times (zero and sixty minutes) by adding 10 *μ*L of 50% H_3_PO_4_. One hundred microliters of each sample were filtered and injected into the HPLC system. The released Ang I peptide was quantified by reverse-phase HPLC using an Aquapore ODS 300 column 7 *μ*m (*PerkinElmer Inc*, EUA) equilibrated with 0.1% phosphoric acid containing 5% acetonitrile (vol/vol). Ang I was separated by isocratic elution for 5 min, followed by a 20 min linear gradient of 5–35% acetonitrile in 0.1% phosphoric acid (vol/vol) at 1.5 mL/min. The chromatographic profile of each sample was compared with that obtained for standard samples containing angiotensinogen and Ang I at an absorbance of 214 nm. Peptide fragments were identified by elution position and quantified by integration area using repeated injections of standard peptide solution to correct for small differences in retention time (<6%) and peak height (<5%).

#### 2.5.2. ACE Activity

ACE catalytic activity was determined fluorimetrically as described by Friedland and Silverstein [27]. An aliquot of kidney homogenate (10 *μ*L) was incubated with a 200 *μ*L assay solution containing 1 mmol/L Z-Phe-His-Leu (ZPhe-HL) or 5 mmol/L HippurylHis-Leu (HHL) in 100 mmol/L borate buffer, with 300 mmol/L NaCl and 0.1 mmol/L ZnSO_4_, for 10 minutes at 37°C. The enzymatic reaction was stopped by the addition of 1.5 mL 280 mmol/L NaOH and incubated with 100 *μ*L o-phthaldialdehyde (20 mg/mL methanol) for 10 minutes. The fluorescent reaction was stopped by the addition of 200 *μ*L 3 N HCl. The liberated dipeptide HL was measured fluorimetrically (360 nm excitation and 500 nm emission) using a Hitachi fluorimeter (Hitachi F-200, Japan). The standard curve was obtained using varying concentrations of L-HL in the blank reaction mixture and it showed a linear relation between relative fluorescence and HL concentration [28].

#### 2.5.3. ACE2 Activity

Renal ACE2 activity was determined in spectrofluorimeter (*Tecan*, Switzerland), using the synthetic fluorogenic substrate, Mca-APK-Dnp (5 mmol/L,), as described by Pedersen et al. (2011) with some modifications [29]. Kidney samples (12 mg) were homogenized in 500 *μ*L buffer containing 75 mM Tris HCl, pH 6.5, 1 M NaCl, and 0.05 mM ZnCl_2_, added by 10 *μ*M of captopril and 1 tablet/10 mL protease inhibitor mixture (Complete, Mini; Roche Diagnostics Corp., Indianapolis, IN). Homogenates were centrifuged twice (15000 rpm, 15 minutes, 4°C) and supernatants were stored at −20°C. To each well, 5 *μ*L of a tissue sample was added, along with 50 *μ*L of buffer in the presence or the absence of ACE2 inhibitor (DX600, 10 *μ*M). Substrate was added and samples were read at 0 and 30 minutes. Arbitrary units were registered, calculations were done based on a fluorescence standard curve (OmniMMP) and the time point 0 was used as internal blank.

#### 2.5.4. Angiotensin Quantification

The extraction of angiotensins was held in Oasis HBL 3cc columns (Waters, Ireland), previously activated with methanol (5 mL), tetrahydrofuran (5 mL), hexane (5 mL), methanol (5 mL), and water (10 mL). After sample introduction, columns were washed with water (10 mL) and peptides of interest were eluted with ethanol, acetic acid, and water (90 : 4 : 6). The eluted fractions were lyophilized and resuspended in 500 *μ*L mobile phase A: 5% ACN in 0.1% orthophosphoric acid and one hundred microliters of each sample were filtered (with 0.7 mm membrane) and then injected into the HPLC system. Peptides were separated by HPLC (Shimadzu System, Japan) in reverse phase column Aquapore ODS 300 (250 mm × 4.6 mm), 7 mm, using a linear gradient from 5% to 35% of mobile phase B (95% acetonitrile in H_3_PO_4_ 0.1%), at a flow rate of 1.5 mL/min for 30 min. The chromatographic profile of each sample was compared with that obtained for standard intact and fragmented peptides (Sigma Co.) of the RAS at 214 nm absorbance. Peptides were identified according to retention time and quantified by area integration using repeated injections of standard peptide solution to correct for small differences in retention time (<6%) and peak height (<5%). The lower limit of quantification determined was approximately 3,125 pmols.

#### 2.5.5. Bradykinin Concentration

Bradykinin levels were measured using commercial available enzyme-linked immunosorbent assay (ELISA) kit (Enzyme-Linked Immunosorbent Assay Kit for Bradykinin, Life Science, USA).

### 2.6. Statistical Analysis

Results are presented as means ± SEM. Results were compared using 2-way analysis of variance (ANOVA) (performed with diabetes and ACE genotype as factors) followed by Tukey's test (or comparisons among multiple groups). Mortality was determined by Kaplan-Meyer survival analyses. A *P* value of <0.05 was considered to be significant. All statistical analyses were performed using the Prism Software package version 5 (GraphPad Software, San Diego, CA, USA).

## 3. Results and Discussion

We compared morphometry and several physiologic and biochemical parameters in renal tissue of normal and diabetic mice bearing 1 and 3 copies of* Ace* gene to uncover the effects of* Ace* genotype, of diabetes, and of any interactions on the development of DN. Our results suggest that* Ace* locus associated with hyperglycemia disrupt RAS balance, generating a complex novel set of peptides synthesis. This includes not only a markedly increase on renal Ang II generation but also high tissue levels of bradykinin and Ang-(1–7), which in turn may have an important contribution in the development of diabetic lesions associated with increased* Ace* gene dosage.

As summarized in Table 1, diabetic levels of plasma glucose were present throughout the 12-week observational period in STZ diabetic mice of both* Ace* genotypes, but not in the untreated controls; glycemia was not affected by* Ace* gene copy number neither in control nor in diabetic groups. As expected, STZ treatment also produced other characteristic signs of diabetes, already described in literature [30] such as increased intake of both water and food and failure to gain weight [31]. Similar results have already been described in this animal model by Huang et al. (2001) and also show that* Ace* genotype does not influence these parameters [21].

**Figure 1:**
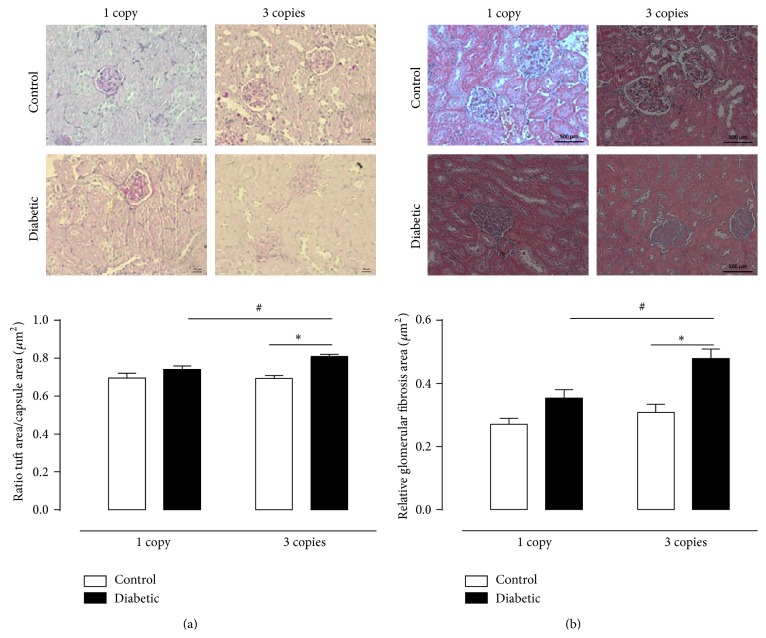
Histological analysis using PAS (a) and Masson's Trichrome stain (b) at 12 weeks of experimental period. (a) Representative photomicrographs depicting PAS staining of kidney sections in control and diabetic mice having 1 or 3 copies of the* Ace* gene. (b) Representative photomicrographs depicting Masson's Trichrome staining of kidney sections in CT and D mice having 1 or 3 copies of the* Ace* gene.

To determine how* Ace* genotypes and diabetes affect patterns of renal morphology and function, we evaluated renal histology, kidney-to-body weight ratio, urinary volume, and also excretion of albumin, creatinine, and urea. It is well known that one of the most striking characteristics of DN is mesangial expansion, which results from accumulation of ECM proteins, decrease in filtration surface, and ultimately end stage renal disease [32–34]. In this study, the glomerular appearance in histology of 3 copies diabetic animals showed accelerated mesangial expansion characterized by an increase in PAS-positive relative mesangial matrix area (Figure 1) compared with that observed in 1 copy diabetic group and also in control groups (3 copies diabetic: 0.808 > 1 copy diabetic: 0.629 > 3 copies control: 0.742 = 1 copy control: 0.692; *P* < 0.05). These alterations in kidney morphology, worse in 3 copies mice, are similar to those seen in the initial stages of human diabetes and contrary to the data presented by Huang et al. (2001) [21]; in this study these kidney structural changes are affected by the* Ace* genotype. Furthermore, Masson's Trichrome staining revealed that glomerular fibrotic areas were significantly increased in 3 copies diabetic mice compared to control mice and also to 1 copy diabetic group (3 copies diabetic: 0.478 > 1 copy diabetic: 0.353 > 3 copies control: 0.308 = 1 copy control: 0.270 *μ* ^2^; *P* < 0.05). To support our findings related to damage in renal morphology induced by increased* Ace* dosage and diabetes, we evaluated kidney hypertrophy. STZ-induced diabetes increases kidney weight gain. This characteristic increase in kidney weight (KW) (measured by hypertrophy index mg of KW/g of body weight) was found in both 1 and 3 copies* Ace* diabetic groups, suggesting renal injury. Renal hypertrophy was significantly higher in 3 copies versus 1 copy diabetic group, showing the important influence of* Ace* gene dosage on this parameter and supporting the data on renal histology.

These differences related to renal morphology reflected alterations in functional capacity of this organ in diabetic animals. Both 1 and 3 copies diabetic groups presented higher urinary volume compared to controls, which consistently increased throughout the study period. Urinary excretion of creatinine and urea, also useful in estimating the extent of impairment of kidney function, was significantly lower in the diabetic groups compared to their controls. Creatinine and urea were not affected by disease duration or genotype.

On the other hand, urinary albumin excretion (UAE), the most commonly used early marker of DN [35], was significantly affected by diabetes and* Ace* genotype. One- and 3 copies control mice have very low levels of UAE which did not change with time. One week after induction of diabetes, neither the 1 copy nor the 3 copies mice have UAE levels higher than their controls. However, at the end of protocol not all diabetic animals presented higher UAE; in other words, only mice harboring 3 copies of* Ace* gene presented greater albuminuria after 12 weeks. Corroborating this result Huang et al. (2001) showed that the UAE of the 1 and 2 copies diabetic mice progresses much less rapidly than 3 copies, so 3 copies mice when diabetic develop overt albuminuria early in the course of the disease [21]. DN is thought to be a unidirectional process from microalbuminuria to end-stage renal failure; however recent studies in type 1 diabetic patients demonstrate that a large proportion of DN patients revert to normoalbuminuria and that one-third of these patients exhibit reduced renal function even in the microalbuminuria stage. This finding (higher UAE presented only by 3 copies diabetic mice) will be thoroughly discussed ahead.

To correlate the structural and functional kidney damage, induced by increased* Ace* gene dosage, RAS components were evaluated.

**Figure 2:**
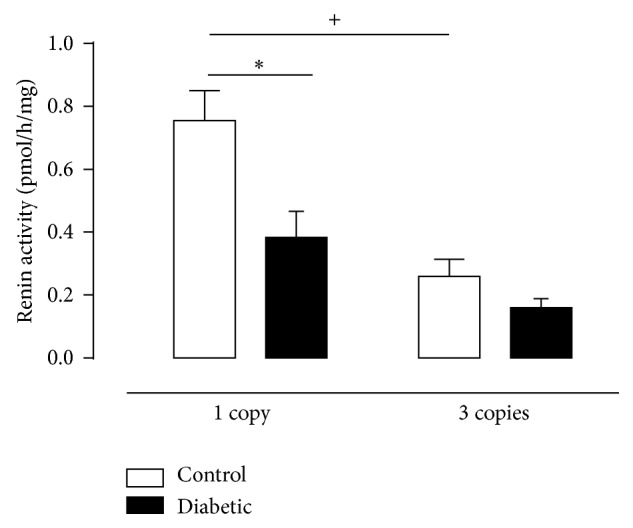
Renin activity in renal tissue of CT (white column) and D (black column) mice having 1 or 3 copies of the* Ace* gene at the end of twelve weeks of experimental period. Renin activity was obtained by incubating renal tissue samples with excess renin substrate for 1 h at 37°C. Results were determined from the amount of Ang I generated in the absence of trypsin by HPLC. Values represent mean ± S.E.M., *n* = 6–11. Two-way ANOVA showed ^*∗*^ *P* < 0.05: diabetic versus control and ^+^ *P* < 0.05: 3 copies control group versus 1 copy control group.

**Figure 3:**
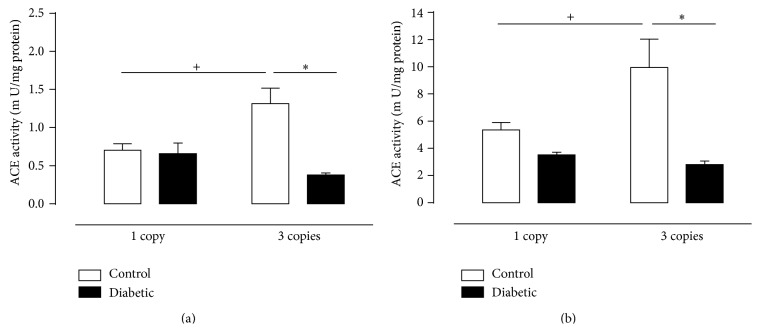
ACE activity (mU/mg of protein) in renal tissue of CT (white column) and D (black column) mice having 1 or 3 copies of the* Ace* gene at the end of twelve weeks of experimental period, assessed using two specific ACE substrates: (a) HHL and (b) Zphe-HL. Values represent mean ± S.E.M., *n* = 6–11. Two-way ANOVA showed ^*∗*^ *P* < 0.05: diabetic* versus* control. ^+^ *P* < 0.05: 3 copies control group* versus* 1 copy control group.

Renal renin activity was estimated with an excess of substrate and is presented in Figure 2 being reduced in diabetic mice having 1 or 3 copies of the* Ace* gene when compared with its controls. Increased* Ace* gene dosage was inversely correlated with ACE activity in control mice; enzyme activity was twice as high in renal tissue from control mice harboring 1 copy of the* Ace* gene, as compared with 3 copies mice. Huang et al. (2001) [21], Evangelista and Krieger (2006) [36], and also Krege et al. (1997) [20] evaluated plasma renin activity in control mice harboring different copies of* Ace* gene and observed an inverse relationship between renin activity or renin gene transcription and* Ace* genotype (activity). Here we also evaluated the effect of diabetes which curiously reduced enzyme activity only in 1 copy group. This is the first report demonstrating influence of diabetes on renal renin activity in this animal model, and the reduction seems to be an important adaption of local RAS to protect the kidney from an excessive generation of Ang II in 1 copy diabetic mice. These findings argue in favor of compensatory adaptations in the RAS to maintain the homeostasis in genetically manipulated animals, and the evaluation of renal ACE activity using HHL (B) Zphe-HL as substrates (Figures 3(a) and 3(b)) also supports these data, since 1 copy control mice present lower ACE activity in the kidney (~50%) as compared with 3 copies ones. We believe that ACE activity was not reduced in 1 copy diabetic animals, as compared with respective controls, because it is already low. In the present study, we also observed a very expressive decrease in ACE activity in renal tissue of 3 copies diabetic mice, as compared to control ones (Figures 3(a) and 3(b)). This compensatory adaptation to probably reduce the generation of Ang II has already been described in other studies using STZ-diabetic mice and also db/db [37, 38].

**Figure 4:**
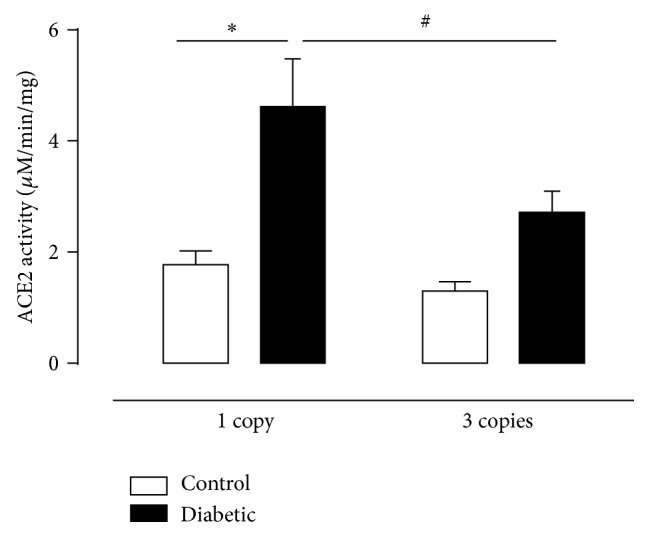
ACE2 activity (*μ*M/min/mg) in renal tissue of CT (white column) and D (black column) mice having 1 or 3 copies of the* Ace* gene at the end of twelve weeks of experimental period. Values represent mean ± S.E.M, *n* = 6–11. Two-way ANOVA showed ^*∗*^ *P* < 0.05: diabetic* versus* control. ^#^ *P* < 0.05: 3 copies diabetic group* versus* 1 copy diabetic group.

The description of ACE2 brought important new data and perspectives to the understanding of RAS regulation, comprised by opposing axes, the classical ACE/Ang II/AT1 and AT2 receptor axis and the ACE2/ Ang-(1-7)/Mas receptor axis [39–41]. The role of ACE2 in the context of diabetes has been widely explored in the past few years. Different studies have shown that decreased ACE2 expression was associated with increased albuminuria [31] and pharmacological inhibition of the enzyme increased urinary albumin excretion three to fourfold in diabetic mouse models [42, 43]. In the present study we observed that diabetes increased ACE2 activity only in 1 copy group, but not in renal tissue of 3 copies animals (Figure 4), and adaptation that has been already described by other groups [42–47]. We detected as presented in Figure 3 decrease of ACE activity in diabetic mice having 1 or 3 copies of the* Ace* gene in contrast with ACE2 activity that was increased in the same animals, suggesting that Ang-(1–7) is derived from both the metabolism of Ang I via the endopeptidase pathway and the cleavage of Ang II by the ACE2-dependent pathway [48].

Taken together, data on albuminuria and ACE2 activity reveals that one of the mechanisms involved on protection against the development of DN present in 1 copy diabetic animals is related to increased ACE2 as already described in other diabetic animal models [43, 49, 50]. In fact, recently studies showed that administration of ACE2 inhibitor (MLN-4760) increases albuminuria, mesangial pathologies, and fibronectin deposition in diabetic mice [38, 42]. In line with these results, Elased [51] observed that urinary ACE2 excretion was positively correlated with the progression of diabetic renal injury represented by progressive albuminuria, mesangial matrix expansion, and renal fibrosis. There is evidence that deletion of ACE2 leads to the development of Ang II dependent renal damage, suggesting ACE2 as renoprotective target in diabetes, most likely a part of a mechanism to compensate for elevated Ang II levels [52–56].

Although some studies describe an increase in ACE2 mRNA levels and protein expression in diabetes [47], Reich et al. (2008) described a decrease in the same parameters in renal biopsies of patients with kidney disease due to diabetes [57] and Colucci et al. (2011) showed a decrease in ACE2 protein expression in the kidney from D-NOD mice [58]. We believe that these differences are related to diabetes duration, and as the progression of disease and DN, the RAS cannot compensate the imbalance on RAS anymore.

**Figure 5:**
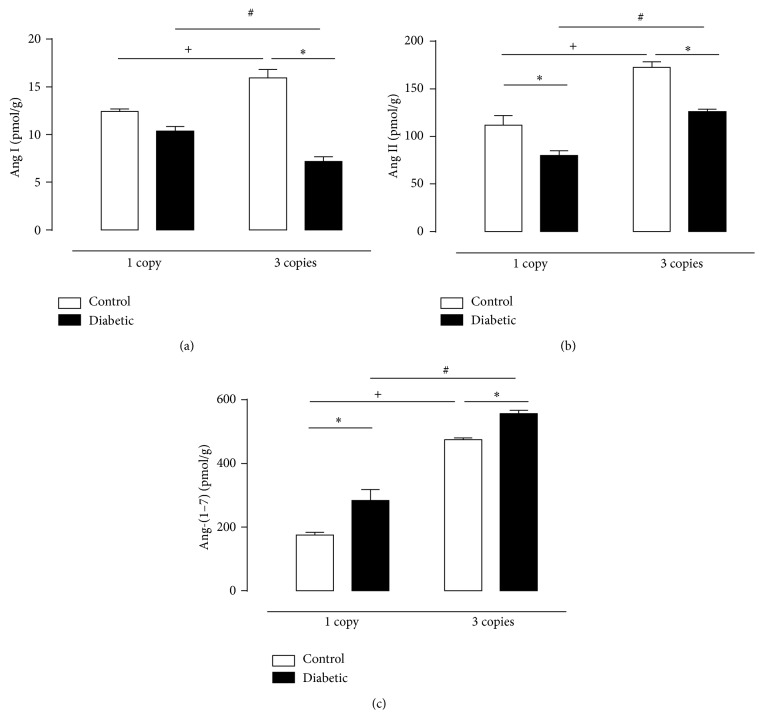
Quantification of (a) Ang I, (b) Ang II, and (c) Ang-(1–7) levels in kidney tissue of CT (white column) and D (black column) mice having 1 or 3 copies of the* Ace* gene at twelve weeks of experimental period assessed by HPLC. Values represent mean ± S.E.M, *n* = 6–11. Two-way ANOVA showed ^*∗*^ *P* < 0.05: diabetic* versus* control. ^+^ *P* < 0.05: 3 copies control group* versus* 1 copy control group. ^#^ *P* < 0.05: 3 copies diabetic group* versus* 1 copy diabetic group.

The impact and the physiological significance of the balance of renal ACE/ACE2 activities cannot be understood without assessing the tissue concentration of angiotensins. Regarding Ang I it is interesting to note that despite the significant reduction in renin activity in 3 copies control mice (compared to 1 copy) our data evidenced a greater increase in the concentration of Ang I in kidney homogenates of this group compared to 1 copy control mice (Figure 5(a) and Table 2). The induction of diabetes did not alter the level of this peptide in mice presenting 1 copy of* Ace* gene; however a significant reduction was observed in 3 copies mice when compared to the control group and to 1 copy diabetic mice. The increase of Ang I presented by 3 copies control mice maybe related to the evidence of Ang-(1–12) being an alternate renin-independent, angiotensin-forming substrate in accordance with data presented by Ferrario et al. [59]. The possibility that Ang-(1–12) may serve as an alternate substrate for the generation of bioactive angiotensins led us to document increased expression of Ang-(1–12) in cardiac myocytes of adult spontaneously hypertensive rats (SHR) compared with Wistar-Kyoto (WKY) controls [59]. Additional studies showed the generation of Ang I, Ang II, and Ang-(1–7) from exogenous Ang-(1–12) in the effluent of isolated hearts from Sprague-Dawley, normotensive Lewis, and mRen2 hypertensive Lewis rats, as well as WKY and SHR [59]. ACE converts Ang-(1–12) to Ang I and Ang II in a sequential reaction consistent with the catalytic properties of the enzyme as a dipeptidyl carboxypeptidase cleaving two residues at a time [60].

As expected, concurrent to increased ACE activity presented by 3 copies control mice, the renal concentration of Ang II was significantly higher in this group compared to 1 copy control mice and to 3 copies diabetic mice (Figure 5(b) and Table 2). Despite the reduction of Ang II induced by diabetes in both groups, the concentration of this peptide was (still) significantly higher in 3 copies mice when compared to 1 copy, so the renal content of this peptide remained 45% higher in 3 copies mice than their control group. So considering, first, as mentioned, that the content of this peptide was higher in 3 copies diabetic mice than 1 copy and, second, that there were no differences between ACE activity from these groups, these results led us to conclude that the generating of Ang II in 3 copies diabetic mice is related to nonclassical pathway.

Although ACE is the most important renal carboxypeptidase that cleaves Ang I at its carboxy terminus to generate Ang II [61, 62] several other described more than three decades ago [63]. Cathepsin G was discovered as an Ang II producing enzyme in human neutrophils [64] and it has been speculated that cathepsin G might be involved in mediating part of the inflammatory response in these cells [65]. Rykl and cols concluded that the angiotensin-generating activities of the fraction containing angiotensin-converting enzyme and the fraction containing cathepsin G were in the same order of magnitude, thus showing that the contribution of cathepsin G towards the production of Ang II is significant [65].

Also chymase is a potent and specific Ang II-forming enzyme in vitro [66] as well in vivo [67]. Furthermore, studies have added that chymase appears to play a role in Ang II formation in certain disease states [68–70], so they observed that evidences indicated that intrarenal Ang II formation is largely mediated by ACE under normal physiological conditions [61, 62, 71], although other non-ACE Ang II-forming enzymes have been recognized in certain types of renal disease [68, 69]. Huang et al. (2003) reported that the chymase-dependent Ang II-generating system was upregulated in the human diabetic kidney and this becomes particularly strong in those with hypertension [68]. In the normal kidney, while ACE was constitutively expressed by most kidney cells, chymase was weakly expressed by mesangial cells (MC) and vascular smooth muscle cells (VSMC) only. In the diabetic kidney, while ACE expression was significantly upregulated (1- to 3-fold) by tubular epithelial cells (TEC) and infiltrating mononuclear cells, there was also markedly increased chymase expression (10- to 15-fold) by both MC and VSMC, with strong deposition in the collagen-rich extracellular matrix including both diffuse and nodular glomerulosclerosis, tubulointerstitial fibrosis, and vascular sclerosis. Interestingly, while ACE expression showed no difference in patients with or without hypertension, upregulation of chymase in hypertensive patients was much stronger than that seen in those without hypertension (4- to 7-fold; *P* < 0.001). Correlation analysis showed that, in contrast to the ACE expression, upregulation of chymase correlated significantly with the increase in BP and the severity of collagen matrix deposition within the glomerulus, tubulointerstitium, and arterial walls (all with *P* < 0.001). In conclusion, the present study suggested that chymase, as an alternative Ang II-generating enzyme, is markedly upregulated in the diabetic kidney and may be associated with the development of diabetic/hypertensive nephropathy. In addition, differential expression of ACE and chymase in the diabetic kidney indicates that both ACE and chymase may be of equal importance for Ang II mediated DN and vascular disease. Results from this study suggest that blockade of both Ang II generating pathways may provide additional beneficial effect on DN [68].

Ang II is the final physiologically active product of RAS, and it works not only as a strong vasopressor but also as a promoter of tissue remodeling in various organs such as heart, arteries, and kidneys [72]. Many lines of evidence suggest a role for intrarenally formed Ang II in the pathogenesis of DN [73, 74]. It has been shown that glucose and Ang II are able to increase the synthesis of collagen types I and IV and other matrix proteins in MC, as well as decreasing the levels of proteases involved in matrix degradation, resulting in the matrix expansion observed in DN [7]. It has been shown that increasing glucose concentrations cause proportional increases in Ang II generation in MC [75].

High glucose concentration induced an impressive elevation of Ang-(1–7) concentration as well as the increasing number of* Ace* gene copies. It is interesting to note that this result (higher levels of Ang-(1–7) presented by 3 copies diabetic mice) (Figure 5(c) and Table 2) has not seem to be due to activation of the classical pathway of the RAS, via hydrolysis of Ang II by ACE2, since ACE2 activity of these animals is reduced. The Ang-(1–7) can be generated by alternative routes involving the hydrolysis of Ang II by prolyl-carboxypeptidase (PCP) and prolyl-endopeptidase (PEP). Further, endopeptidases, such as neprilysin (NEP) or PEP, can also cleave Ang I to generate Ang-(1–7) [76] and, in fact, our results indicate that this maybe the production route used (synthesis of Ang-(1–7) from Ang I by hydrolysis of the NEP and PEP) since the evaluation of the concentration of this peptide (Ang I) is significantly reduced in the diabetic group 3 copies related to all other groups. We cannot exclude the processing also of Ang (1–12) specially in renal cortex to Ang-(1–7) formed by neprilysin [60].

Although Ang-(1–7) is usually described as antiproliferative and antifibrotic, opposing most of the effects of Ang II, it has been demonstrated that it can worsen renal dysfunction in experimental models triggering hypertrophy of this organ. Zimpelmann and Burns (2009) noted that the stimulation of p38 MAPK phosphorylation by Ang-(1–7) leads to release arachidonic acid and production of TGF-beta1 and extracellular matrix proteins led them to conclude that Ang-(1–7) exerts growth-stimulatory effects in human MC [13]. Simões e Silva et al. (2006) observed that end stage renal disease subjects exhibited a dramatic increase in Ang-(1–7) (25-fold higher than control values) and added that it is yet unknown if the elevation of Ang-(1–7) occurs as a compensatory mechanism that opposes the harmful renal and cardiovascular effects of Ang II or whether, due to a major unbalance in the RAS metabolism, Ang-(1–7) at supra physiologic concentrations could act as a mediator of renal dysfunction [77]. Esteban et al. (2009) showed that renal deficiency for Mas diminished renal damage in models of renal insufficiency as unilateral ureteral obstruction and ischemia/reperfusion injury while the infusion of Ang-(1–7) to wild-type mice pronounced the pathological outcome by aggravating the inflammatory response [14]. Recently, Velkoska et al. (2011) concluded that their results add to the increasing evidence that Ang-(1–7) may have deleterious cardiovascular effects in kidney failure and highlight the need for further in vivo studies of the ACE2/Ang-(1–7)/Mas receptor axis in kidney disease [78]. Then, one of the most important events observed in the present study was an impressive rise in Ang-(1–7) content induced by high-glucose exposure and increasing number of* Ace* gene copies, suggesting that the high concentration of this peptide may be associated to increased renal damage presented by 3 copies diabetic mice.

**Figure 6:**
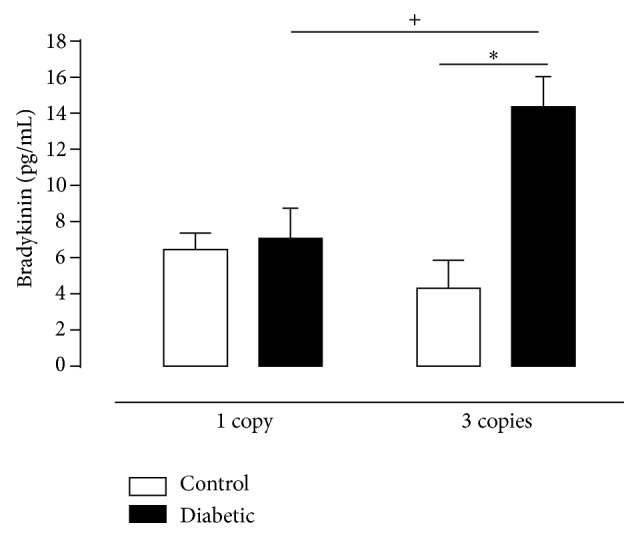
Bradykinin concentrations in kidney homogenates of CT (white column) and D (black column) mice having 1 or 3 copies of the* Ace* gene at twelve weeks of experimental period assessed by* ELISA.* Values represent mean ± S.E.M, *n* = 6–11. Two-way ANOVA showed ^*∗*^ *P* < 0.05: diabetic* versus* control. ^+^ *P* < 0.05: 3 copies control group* versus* 1 copy control group.

Bradykinin level in renal tissue (Figure 6) did not differ from diabetic to control 1 copy mice, however when the diabetes was associated to 3 copies of* Ace* gene, a greater increase of this peptide was observed, according to reduced ACE activity in this group. It is well known that bradykinin is inactivated by ACE, and indeed the 3 copies diabetic animals showed a significant reduction of enzyme activity. Campbell et al. (1999) also found increased levels of bradykinin in the renal tissue of rats treated with STZ [79]. However, Colucci et al. (2011) observed an increase in bradykinin concentration, concomitant with increased ACE activity in the same tissue in NOD hyperglycemic animal model, suggesting that the increase was caused by higher activity of kallikrein, rather than by alterations in the metabolism of kinins by ACE [58, 79]. Moreover, it is important to note that the increase in renal tissue bradykinin observed in 3 copies diabetic animals may also contribute to worsening renal function displayed by these animals, as well as by increasing the mortality rate, since this peptide is involved in vascular and metabolic dysfunctions induced DM [79].

## 4. Conclusion

The susceptibility to the development of DN in 3 copies animals is associated with an imbalance in activity of various enzymes (renin, ACE, and ACE2) leading to increased synthesis of Ang II and Ang-(1–7). Increased renal concentration of Ang-(1–7) appears to potentiate the deleterious effects triggered by Ang II on renal structure and function. Apart from the involvement of the RAS in the development of DN, our results also show increased bradykinin concentration only in 3 copies of the* Ace* gene diabetic group. Taken together, these adaptations indicate that the deleterious effects described in 3 copies of the* Ace* gene diabetic group are, at least in part, due to a combination of factors not usually described in the scientific literature. Thus, the data presented here show up as innovative and contribute in understanding the complex mechanisms involved in the development of DN, in order to optimize the treatment of patients with this complication.

## Figures and Tables

**Table 1:** Main physiologic and biochemical parameters of control and diabetic groups at first week and 12 weeks after induction of diabetes.

Parameter/group	First week of experimental protocol				Twelfth week of experimental protocol			
	1 copy		3 copies		1 copy		3 copies	
	Control	Diabetic	Control	Diabetic	Control	Diabetic	Control	Diabetic
Body weight (g)	24.6 ± 0.1	26.3 ± 0.5	25.1 ± 0.8	26.8 ± 0.7	28.5 ± 0.6	23.7 ± 1.7^*∗*^	30.0 ± 1.1	25.1 ± 0.9^*∗*^
Plasma glucose (mg/dL)	113 ± 5	348 ± 17^*∗*^	106 ± 4	355 ± 30^*∗*^	132 ± 7.30	499 ± 20^*∗*^	133 ± 3	459 ± 29^*∗*^
Food intake (g/24 h)	2.5 ± 0.2	4.5 ± 0.2^*∗*^	3.50 ± 0.4	4.2 ± 0.4^*∗*^	2.8 ± 0.4	4.7 ± 0.4^*∗*^	2.9 ± 0.2	6.1 ± 0.3^*∗*^
Water intake (mL/24 h)	4.6 ± 0.5	17.7 ± 1^*∗*^	5.4 ± 0.3	14.0 ± 2.8^*∗*^	4.0 ± 0.6	17.6 ± 3.8^*∗*^	5.9 ± 0.8	25.7 ± 2.5^*∗*^
Urine volume (mL/24 h)	0.6 ± 0.1	10.8 ± 1.3^*∗*^	1.3 ± 0.1	11.8 ± 2.2^*∗*^	0.7 ± 0.1	13.4 ± 2.9^*∗*$^	1.1 ± 0.2	20.6 ± 2.8^*∗*$^
Relative kidney weight (g/g × 1000)	—	—	—	—	5.8 ± 0.2	8.6 ± 0.5^*∗*^	5.6 ± 0.2	7.2 ± 0.2^*∗*#^
Albuminuria (mg/24 h)	1.1 ± 0.2	1.7 ± 0.9	1.7 ± 0.2	2.4 ± 0.1	1.4 ± 0.2	2.1 ± 0.8	1.7 ± 0.3	4.9 ± 1.0^*∗*#^
Urinary urea (mg/24 h)	49 ± 1	18 ± 1^*∗*^	55 ± 1	20 ± 1^*∗*^	45 ± 3	20 ± 1^*∗*^	49 ± 10	15 ± 2^*∗*^
Urinary creatinine (mg/24 h)	0.40 ± 0.01	0.09 ± 0.01^*∗*^	0.38 ± 0.03	0.04 ± 0.01^*∗*^	0.38 ± 0.06	0.08 ± 0.01^*∗*^	0.39 ± 0.12	0.06 ± 0.01^*∗*^

Values represent mean ± S.E.M., *n* = 6–11. Two-way ANOVA or two-way ANOVA for repeated measures (whether necessary) showed ^*∗*^ *P* < 0.05: diabetic versus control, ^#^ *P* < 0.05: 3 copies diabetic group versus 1 copy diabetic group, ^$^ *P* < 0.05: versus same diabetic group at first week.

**Table 2:** Quantification of Ang I, Ang II, and Ang-(1–7) levels in kidney tissue of CT and D mice harboring 1 or 3 copies of the *Ace* gene at twelve weeks of experimental period assessed by HPLC.

Peptide concentration (pmol/g)	1 copy		3 copies	
	Control	Diabetic	Control	Diabetic
Ang I	12.4 ± 0.3	10.4 ± 0.5	15.9 ± 0.9	7.2 ± 0.5^*∗*#^
Ang II	106.9 ± 9.5	85.1 ± 6.1^*∗*^	172.6 ± 6.0^+^	126.5 ± 2.2^*∗*#^
Ang-(1–7)	174.9 ± 8.2	283.7 ± 34.0^*∗*^	474.6 ± 5.5^+^	556.8 ± 10.9^*∗*#^

Values represent mean ± S.E.M. Two-way ANOVA showed ^*∗*^ *P* < 0.05: diabetic versus control. ^+^ *P* < 0.05: 3 copies control group versus 1 copy control group. ^#^ *P* < 0.05: 3 copies diabetic group versus 1 copy diabetic group.
